# Self-management support in flemish primary care practice: the development of a preliminary conceptual model using a qualitative approach

**DOI:** 10.1186/s12875-022-01652-8

**Published:** 2022-03-31

**Authors:** Lotte Timmermans, Dagje Boeykens, Mustafa Muhammed Sirimsi, Peter Decat, Veerle Foulon, Ann Van Hecke, Mieke Vermandere, Birgitte Schoenmakers, on behalf of the Primary Care Academy, Roy Remmen, Emily Verté, Muhammed Mustafa Sirimsi, Peter Van Bogaert, Hans De Loof, Kris Van den Broeck, Sibyl Anthierens, Ine Huybrechts, Peter Raeymaeckers, Veerle Buffel, Dirk Devroey, Bert Aertgeerts, Birgitte Schoenmakers, Lotte Timmermans, Veerle Foulon, Anja Declerq, Dominique Van de Velde, Pauline Boeckxstaens, An De Sutter, Patricia De Vriendt, Lies Lahousse, Peter Pype, Dagje Boeykens, Ann Van Hecke, Peter Decat, Rudi Roose, Sandra Martin, Erica Rutten, Sam Pless, Anouk Tuinstra, Vanessa Gauwe, Didier Reynaert, Leen Van Landschoot, Maja Lopez Hartmann, Tony Claeys, Hilde Vandenhoudt, Kristel De Vliegher, Susanne Op de Beeck

**Affiliations:** 1 Academic Centre of General Practice, KU Leuven, Kapucijnenvoer 7, Box 7001, 3000 Leuven, Leuven, Belgium; 2 Department of Rehabilitation Sciences, Ghent University, Ghent, Belgium; 3 Department of Public Health and Primary Care, Ghent University, Ghent, Belgium; 4 Centre for research and innovation in care, University of Antwerp, Antwerp, Belgium; 5 Department of Primary Care, University of Antwerp, Antwerp, Belgium; 6 General Practice and Primary Health Care, Ghent University, Ghent, Belgium; 7 Clinical Pharmacology and Pharmacotherapy, KU Leuven, Leuven, Belgium; 8 Centre for Nursing and Midwifery, Hecke University, Ghent University, Ghent, Belgium; 9 Department Nursing Director, Ghent University Hospital, Ghent, Belgium; 10 Academic Centre of General Practice, KU Leuven, Leuven, Belgium

**Keywords:** Self-management, Patients, Primary health care, Health personnel, Qualitative research

## Abstract

**Background:**

Coping with a chronic disease can be really challenging. Self-management represents a promising strategy to improve daily life experiences. The role of primary healthcare professionals cannot be underestimated in supporting self-management. Due to a shortage of theory, implementation of self-management support is hindered in primary care practice. The aim of this study is to create a conceptual model for self-management support by analysing patients’ care experiences towards self-management support.

**Methods:**

An explorative-descriptive qualitative study was conducted in Flanders, Belgium. Semi-structured interviews were performed with 16 patients and their informal caregiver (dyads) using a purposive sampling strategy and processed by an inductive content analysis, according to Graneheim and Lundman.

**Results:**

Interviews revealed in-depth insights into patients’ care experiences. A conceptual model was developed for primary care practice, including five fundamental tasks for healthcare professionals - Supporting, Involving, Listening, Coordinating and Questioning (SILCQ) – contributing to the support of self-management of chronic patients.

**Conclusions:**

This qualitative paper emphasises the use of the SILCQ-model to develop optimal roadmaps and hands-on toolkits for healthcare professionals to support self-management. The model needs to be further explored by all stakeholders to support the development of self-management interventions in primary care practice.

**Supplementary Information:**

The online version contains supplementary material available at 10.1186/s12875-022-01652-8.

## Background

There is a growing number of people worldwide living with multiple chronic conditions [1, 2]. Approximately one on three adults has to face the challenges of living with multimorbidity [3]. In Europe, this number of people is estimated at more than 50 million [4]. Chronic conditions are defined by the World Health Organization (WHO) as long standing and slowly deteriorating diseases [5]. Due to this chronic character, the impact on a patient’s daily life cannot be underestimated.

The consequences of chronic diseases are generally reflected in limited capacity, reduced functionality and productivity, reduced quality of life and increased healthcare costs [6, 7]. As a result, chronic diseases require intensive management. Multiple interventions have been developed in order to support people with chronic diseases [7–9]. Key roles are mainly reserved for the healthcare professionals and the social environment of the patient [10–14] but even more important in the care process is the role of the patient himself [15]. By taking charge of their own chronic diseases, patients fully commit in their own care [16]. This responsibility helps them to function in daily life activities, to experience interdependency throughout the care process, and offers the possibility to optimize patient’s own care [17]. In addition, taking charge of their own health contributes to the development of self-management [18].

Self-management is defined as “the individual’s ability to manage the symptoms, treatment, physical and psychosocial consequences and lifestyle changes inherent in living with a chronic condition” (Barlow et al. 2002) [19]. Patients can be assisted in this process of taking ownership by healthcare professionals. Health care systems are gradually changing as to support these professionals in taking up this role. This change is defined as “the process of making and refining multi-level changes in healthcare systems (and the community) to facilitate patients self-management” (Glasgow et al. 2003) [20]. Self-management support implies intensive cooperation among patients, healthcare professionals and the healthcare system [21]. Primary healthcare professionals are well-positioned to help patients developing the ability of self-management, since primary care often serves as the first point of contact in the care system [22]. They have the opportunity to encourage patients to take part in their own care process. As a result, patients feel empowered and can actively be involved in their health care [23–25].

Several models have been developed to guide self-management support interventions for patients with chronic conditions. For example, the WISE-model (Thompson et al., 2018) aims to support patients by focussing on active counselling by trained healthcare practitioners [26]. The model seeks to encourage practices to incorporate patient-centred care. The five A’s Model (Glasgow et al., 2003) also focusses on patient counselling [20]. In this model the creation of a personal action plan informed by 5 A’s elements (i.e., assess, advise, agree, assist and arrange) is central, which is considered as essential to facilitate patient self-management [20]. Various other approaches to self-management support are presented in literature. In 2017, the strategic international chronic condition self-management support framework (Mills et al.) formulated guiding principles and strategic directions prior to the establishment of self-management support initiatives [27]. A comparative overview for different support frameworks exists and aims to create a platform for researchers to operationalise frameworks (O’connell et al., 2018) [28].

Although they are designed to be applicable in primary care practice, self-management support interventions are not yet optimal in use and their effectiveness is questioned [29–32]. The models and frameworks described in literature tend to focus on the implementation of self-management support rather than on underlying mechanisms, such as the interaction between healthcare professionals and their patients. However, understanding these mechanisms is of great importance in effective self-management support [33].

In addition, existing models are drawn up by researchers based on cooperation with healthcare professionals, instead of with patients. Since patients act as equal partners in a collaboration on shared responsibility [34], the importance of involving them in research cannot be underestimated. More insights are required to explore patients’ experiences towards this partnership to self-manage a chronic condition [25]. Therefore, there is a need to investigate not only the underlying mechanisms that help people to self-manage a chronic condition, but also to listen to the voice of the patients themselves. These understandings should supplement insights from existing models and orientate the further development of new self-management support interventions.

This study offers an exploratory view on patient’s self-management and on the support by primary healthcare professionals. The aim is to create a conceptual model for self-management support by analysing patients’ experiences. The research question addressed in this study is, “What do we learn from patients’ care experiences about the interaction with care professionals related to self-management support?”. This study is part of a larger research project of the Primary Care Academy (PCA) that aims to explore patients’ experiences towards primary care in Flanders (Belgium).

## Methods

### Study design

This qualitative study explored experiences of chronic patients and their informal caregivers, so called dyads, with primary healthcare in Flanders. The aim of the study was to explore specific experiences regarding self-management support by healthcare professionals. For this purpose, a qualitative content approach was used to analyse the transcripts (Graneheim et al., 2017) [35].

### Study participants

The dyads were purposively sampled. The inclusion criteria applied to the patients and were defined based on the definition of patients with complex care needs operationalized by Iglesias [36] and adapted to the specific research question. We did not set out specific criteria for the informal caregivers.

#### Inclusion criteria

The interviewed patients were purposively recruited and had to meet all the following criteria: (1) aged 18 years or above, (2) suffering from a single severe chronic condition or two or more stable chronic conditions (defined as multimorbidity), and (3) receiving support from at least three primary healthcare disciplines, in addition to the support of an informal caregiver.

To achieve a heterogeneous maximal variation sample, the patients had to meet one of the following additional criteria: (1) taking four or more different medications related to their chronic condition(s), (2) demanding a higher need of care, (3) living in a low socio-economic situation, (4) estimated to have limited or low health literacy, or (5) tending to need more care according to at least one member of their primary care team.

#### Exclusion criteria

Exclusion criteria were defined for either ethical or practical reasons and assessed by the entire research team: (1) patients legally incapacitated to participate, (2) patients incapable to reason about care for various reasons (e.g., severe mental illness, cognitive impairment), (3) patients incapable of being interviewed during the predetermined time frame, (4) patients unable to give permission by the informed consent form, and (5) patients with terminal illness.

Dyads were approached by health and welfare organisations or by their General Practitioner (GP), who provided general information on the study. Oral permission of the dyads to their GP was required for the research team to contact the participants. The researcher explained the informed consent form and provided additional information on the project. The dyads were assured participation was completely voluntary and their informed consent was obtained before the beginning of the interviews.

### Data collection

Semi-structured interviews were organised with dyads in their home setting or due to the COVID-19 pandemic by using video conferencing platforms. The interviews were conducted between January 2020 and August 2020 and were supported by an open-ended interview guide. Questions focused on patients’ primary care experiences and their interaction with healthcare professionals. More specifically, open-ended questions in the interview related to (1) daily experiences of living with a chronic condition; (2) structure and functioning of the care network; (3) empowerment, involvement, and participation in care processes; (4) needs, goals and wishes towards primary care and (5) guidance and support strategies in primary care. Every interview was scheduled to last no more than one hour a half. The interviews were addressed to the patient, but the informal caregiver was offered the possibility to add input or support the conversation to improve patient understanding. The collection of data was pilot tested, and the first interviews were conducted by two researchers of the team (DB, MS, and LT) together. The subsequent interviews were conducted independently by one researcher of the same team, individually recorded and transcribed verbatim.

### Data analysis

To address the research question in this paper, a qualitative inductive content analysis was undertaken by the main researcher LT (Graneheim et al., 2017) [35]. First, transcripts were read multiple times to gain an overall naive understanding. Afterwards, the data were reduced into meaningful units addressing patients’ self-management support experiences. Subsequently, units were condensed and labelled with codes. Thereafter, the codes were compared and allocated into subcategories by classifying the codes according to similarities and differences. Similar subcategories were merged into each other and categorized into main categories. Finally, a reverse approach was used, and the overall main categories were tailored to the initial data. Data analysis was conducted using an Excel spreadsheet. Meaningful units, condensations, subcategories, and categories were checked by the principal investigator (BS) and confirmed to increase the credibility of the results. We considered data saturation as the end point of data collection and analysis, meaning no new data were generated from the interviews.

### Trustworthiness

To prove trustworthiness of the data, the criteria of credibility, transferability, dependability and confirmability were applied in this study (Guba and Lincoln) [37]. To enhance credibility of the analysis, we used member-checking to ensure accuracy. Member-checking of the findings was performed by presenting the model and findings to a minority of participants. In addition, maximum variation sampling was applied to recruit the interview participants. To increase the transferability, participants of different age, socioeconomic level, educational level, living and health status were chosen. Moreover, we tried to ensure this transferability of the data by including direct quotations in the manuscript. To enhance dependability of our data, the interviews were carefully recorded and transcribed verbatim. In addition, the analysis was checked by qualitative experts in the field, which has also contributed to the confirmability of our data. The latter criterion was further increased by including appropriate samples with maximum variation.

### Research team

The interviews were collected by DB, MS, and LT; the analysis of the experiences towards self-management support was performed by the main researcher LT in close collaboration with the entire research team. Before the project initiation, the team received a training on the principles and methods in qualitative research to assure a certain level of standardization.

### Ethics

Ethical approval for the original study was obtained from the Ethical Committee of University of Antwerp (B300201942302). All methods were carried out in accordance with relevant guidelines. The entire study was in accordance with the Helsinki Declaration.

## Results

In total, 16 interviews with a patient-informal caregiver dyad were performed. The interviews lasted from 58 to 90 min. Although the interviews were conducted with patients in the presence of their informal caregivers, the results presented in this paper entirely focus on the patient’s experiences towards self-management support. Involving the informal caregivers helped patients in formulating their story and to feel at ease.

### Characteristics of study participants

A total of 32 persons were interviewed, including 16 patients and 16 informal caregivers. Most patients were living together with their informal caregiver (11 out of 16 patients). The characteristics of the participants are summarized in Table 1.

**Table 1:** Characteristics of the participants

	Patients	Informal caregivers
Gender		
Male	**5**	**7**
Female	**11**	**9**
Mean age	**67.5**	**66.8**
Additional inclusion criteria		
Patients taking four or more different medications	**11**	**-**
Patients demanding a higher need of care	**6**	**-**
Patients of low socio-economic situation	**3**	**-**
Patients estimated to have limited or low health literacy	**3**	**-**
Patients tending to need more care according to at least one member of their primary care team	**2**	**-**
Employment		
Employed	**0**	**1**
Unemployed	**0**	**1**
Unemployed due to disability	**3**	**3**
Retired	**13**	**11**

### Structural analysis

The inductive content analysis of the interview data resulted in five main categories regarding the role of healthcare professionals in reinforcing patients’ self-management: supporting, involving, listening, coordinating, and questioning. The main categories are the result of breaking down the interview transcripts into meaning units, further condensed into multiple subcategories, and finally encapsulated into main categories (Table 2). In some cases, a subcategory applies to multiple main categories since the main categories are not strictly delineated and overlap slightly. Table 3 provides an overview of the main categories and subcategories. The categories exclusively focus on interactions between patients and their healthcare professionals.

The healthcare professional network consisted in many cases of the same key actors including a GP, a pharmacist, a home nurse, and a medical specialist related to a specific disease. Typically, the primary care professionals acted as the first and central point of contact for patients. Depending on patients’ case, other primary healthcare professionals were also involved like social workers, physiotherapists, speech therapists, members of the pain clinic, dietitian, etc. A patient with Parkinson’s disease, for example, would be additionally supported by a physiotherapist and a speech therapist. In a limited number of interviews, there was also guidance by a psychologist or psychiatrist. In the following section, the different categories are presented with examples of verbatim quotes.

**Table 2:** Example of the inductive content analysis

Meaning unit	Condensation	Subcategory	Main Category
*“She [GP] helped me… I can always go to visit her or call if I do not feel okay. Not for the prescription of my medications, but to meet for a talk.”* (Patient – P4)	The doctor is both physically and by telephone accessible for not only the prescription of medication, but also for a listening ear.	Accessibility Understanding	Coordinating Listening
*“I have balance problems. If the physiotherapist comes at home, our first exercise is on it.” … “We walk around. She stands behind me when she sees me shaking so she can quickly grab me.”* (Patient – P2)	Treatment of the physiotherapist with supportive exercise (balance problems)	Practical support Formal support	Supporting
*“When I was first diagnosed, I had many questions. And I gave them to him [doctor].” … “That doctor just answered the questions he wanted to respond. And when the doctor’s appointment was over, I never received the answers on the questions, he did not likely formulate a response on. So yes, that was a mess … and I did not feel good about it.”* (Patient – P6)	Patient used to write the questions down before an appointment. The doctor just answered the questions he wanted to answer and then the appointment was over. Patient didn’t feel good about it.	Dialogue Information	Questioning

**Table 3:** Overview of subcategories and main categories resulting in an overall core category

Subcategory	Main category	Core category
Practical support ADL support Physical support Household support Medical support Information exchange Clinical expertise Follow-up	Supporting	The role of healthcare professionals in self-management support
Communication tools Shared decision-making Participation Cooperation Care continuity Freedom of choice	Involving	
Taking time Empathy Understanding Listening ear Dealing with help requests Emotional support Listening to questions Listening to expectations Listening to wishes and goals Listening to care barriers and facilitators	Listening	
Accessibility Care continuity Deliberation Stability Collaboration Time management Support network Teamwork Point of contact Follow-up	Coordinating	
Dialogue Information Questioning expectations Questioning experiences Questioning wishes and goals Questioning care barriers and facilitators	Questioning	

#### Supporting

Within our study context, we defined supporting as all elements supplied by a healthcare professional related to treatment, follow-up, and guidance. The participating patients indicated to be accompanied by a team of healthcare professionals. According to the interview data, the main actors involved in the active support were nurses, GPs, and physiotherapists. Their support was experienced as essential to fit the chronic condition into daily life. In essence, essential to self-manage the disease.

*“For my care… I don’t think other persons are involved beside my physiotherapist, the home nurse and my GP.” (patient)*

Besides delivering physical care (e.g., diagnosis, treatment, symptom control), various other support elements came up in the interviews. More specifically, supporting was about actively guiding through the delivery of practical tools (e.g., a wheelchair), health-related information, medical assistance (e.g., medicines) and the set-up of home care. Home health care represented most of the support. The frequent visits by home care nurses ensured a better disease management.

*“And then in the evening, the physiotherapist comes to visit at home. And he provides exercises for my back and my neck and he applies a bandage with a tape." … “It really helps me. He supports me well.” (patient)*

*“The purpose of the nursery is to make the physical condition as good as possible.*

*That goal has been realized.” (patient)*

#### Involving

Involving was defined as working in (co-)partnership with patients. Participants described explicitly the wish to be involved in their care. Patients experienced feelings of respect and equality when being actively engaged in health care. It gave them a chance to participate in their own care and to self-manage their chronic condition. The involvement seemed to be mostly related to medical decision making. For example, patients longed for a freedom of choice related to medical treatment options. In addition, the wish was expressed to be involved in decisions concerning the support of daily life activities. Well-informed decisions did arise when there was room for open conversation and discussion. The extent to which patients wanted to be involved depended on the patient.

*“If I have an appointment with D. [the doctor], he asks me if I want to try the proposed medicine or if I prefer something else. So yes, I’m taking part in the decisions he makes.” (patient)*.

Moreover, patients strived for involvement throughout the entire care process. To overcome the pitfall of a provider-centred management, patients indicated empathy and understanding as essential components of good collaboration. The participants expressed the wish to share their concerns to feel comfortable in the care they receive.

*“No, I make decisions together. Uh, for example… They have asked me a couple of times what I think about a DBS [Deep Brain Stimulation]. Uh, I am quite reluctant towards it*

*and therefore, I have made it clear to the doctor.” (patient)*

#### Listening

Listening seemed one of the most talked-about components, defined as the act of giving an audience and paying attention to someone. Patients expected from their healthcare professionals to listen to what they need, what they want and to what they strive for. Only in this way patients felt supported to manage the chronic disease. In addition, the interviews emphasised the importance of encountering a professional with a listening ear to be able to express concerns. The interview participants pointed towards the home care nurse, a psychologist, or the GP as the ideally positioned sounding board.

*“She [GP] helped me… I can always go to visit her or call if I do not feel okay.*

*Not for the prescription of my medications, but to meet for a talk.” (patient)*

Listening to patients was defined as taking time and being into an accessible mindset. Furthermore, listening means patients felt heard. Participants clearly mentioned that plenty of opportunities can arise from open-minded interactions between care professionals and care receivers.

*“You have the feeling that those doctors are making time for you. It’s not that you walk in their practice and they immediately start looking at the clock… If the doctor comes here for a home visit, and he always comes here, he’ll just sit down for half an hour, at least, and tell you all kind of things. He is also a nice person… and he listens to you and gives advice.” (patient)*

The listening aspect was also mentioned in the interviews when talking about emergency situations. Patients were in need to contact their care professionals if they experienced problems. Dealing with help requests included active listening to all actors involved. The GP mostly acted as the central contact person in these cases.

#### Coordinating

Coordinating was defined as guiding responsibilities in the entire care process. According to the participants, coordinated care contributed to coping with their chronic condition, and consequently, to self-manage the chronic condition. Patients expected healthcare professionals to assume responsibility to keep the care network running. An effective follow-up was fundamental by means of coordination between the professionals.

Furthermore, patients expressed the importance of being able to make an appeal to their formal network. Again, the central connection was the patients’ GP.

*“They work well together. P. [GP1] is the one who coordinates everything a bit. J. [GP2] has been away for a year because she had a baby. P. and J. used to come around. During the holidays we received a letter that someone is stepping in. They are all good doctors. They coordinate with each other very well.” (patient)*

According to the participants, the act of coordinating involves communication and discussion between different actors through the entire care process. Participants experienced benefits of information exchange since it guaranteed care continuity. In addition, coordinating included collaborating among the healthcare professionals with a focus on the chronically ill patient. This could be facilitated using digital tools (e.g., digital patient file, electronic health platforms). The interview conversations highlighted the significance of a well-functioning structured care system, in which effective and clear agreements are made. Finally, coordinating also meant that there was good overview of all care actors involved.

*“Doctor X [GP1] and doctor Y [GP2], they come here for a home visit. And they have their computers with them. They contain the entire patient file. Everything is written inside that has happened. And if someone [one of the two central GPs] cannot come to us, there are always others doctors available. You see, that’s how our doctors are here.” (patient)*

#### Questioning

For the current analysis, we defined questioning as a type of communication giving raise to conversation by using interrogation. Patients expected the care professionals to ask them questions. Posing questions created a genuine momentum between care professionals and patients. It resulted in an interactive conversation about patient’s wishes, goals, and expectations. Furthermore, formulating questions to the patient initiated valuable conversations about the care process: what goes well and what could be improved? Do patients feel comfortable in their care, do they understand the medical treatment or are there any ambiguities? Additionally, a question could boost the mechanisms of information transfer between patients and their care professionals.

*"A great healthcare professional takes time, poses question and talks." (patient)*

## Conceptual model

**Fig. 1:**
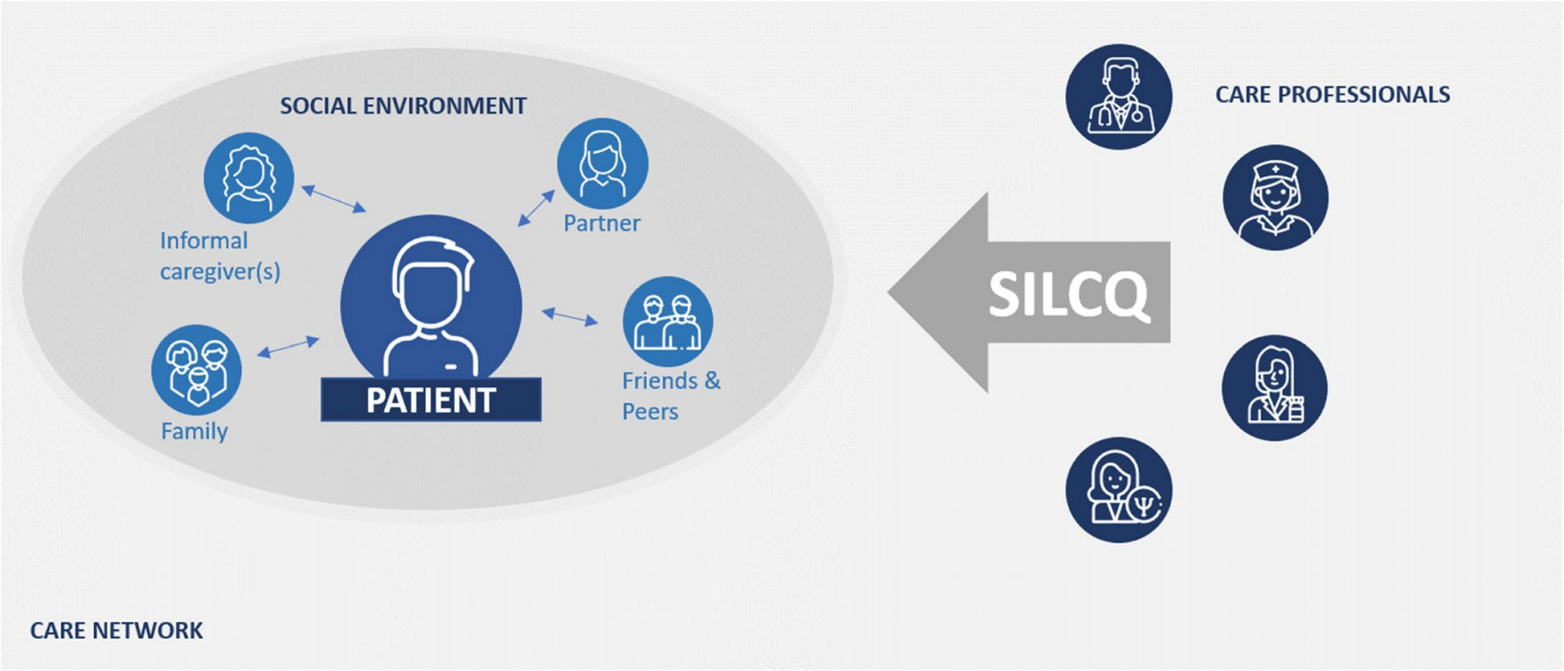
Conceptual model on the support of self-management. Abbreviations: S = Supporting; I = Involving; L = Listening; C = Coordinating; Q = Questioning. Icon made by Freepik from www.flaticon.com

Using in-depth interviews, insights into patients’ primary care experiences related to self-management support were gained. We learned that patients could manage their chronic disease more effectively if they feel supported, involved in the care process, listened to, if their care is coordinated and if they are questioned. Based on these five main categories, we were able to identify and formulate concepts to describe the key characteristics in the support of self-management. The conceptual model for primary care practice includes five fundamental tasks that need to be performed by healthcare professionals - Supporting, Involving, Listening, Coordinating and Questioning (SILCQ) – that contribute to efficiently supporting chronic patients’ self-management. These tasks were incorporated into the model as the acronym ‘SILCQ’ (Fig. 1).

Patients are in most cases connected to a social network. We defined this network as ‘the social environment’. According to the participants, the social environment consists of the closest surroundings of a patient. The composition of the social environment varied and was different depending on the patients. Possible members were relatives, friends, and partners. This network was reinforced by peers in some cases. In this close environment, someone had taken upon the role of informal caregiver.

*“I am in contact with the health insurance fund. Also, with my parents, of course, and my friends and family… And the nurses for appointments and to accompany me to scans etc. And my home help… And the family doctor.” (patient). *

*“I think that fellow sufferers are important. Like the pain society…. That’s very good because there you get to know people who also understand you and who are also going through the same thing to some extent. I find the support of those people enormous.” (patient)*

Being able to manage a chronic condition is closely related to support by the entire care network, including the social environment. The proposed model considers these interactions in a patient-centred care network.

*“And if she suddenly has a disease flare, I call the medical practice to ask for help. Afterwards, I can get a medicine from the pharmacist, because in those situations she is out of medication. When I call, the doctor says: ‘Yes, go ahead.‘ So, I call the pharmacist and I say to them: ‘I have a problem, but I don’t have the prescription for the medication yet. Can I already pick up the medication?‘ I’ll bring it later, and that is not a problem.” (informal caregiver).*

## Discussion

The findings highlight healthcare professionals’ role in supporting self-management of chronic conditions. These roles are reflected in the proposed SILCQ-model. Unfortunately, important components such as arranging follow-up are often ignored in practice [38]. Due to the high demands for time and attention, the focus during clinical encounters is mainly on the current problems of patients [39]. The adoption of follow-up with healthcare professionals is additionally challenged because it may be difficult for patients to understand the concept or may be considered an unacceptable intrusion into daily life [40]. Nevertheless, our study emphasises the importance of active support from healthcare professionals during both treatment and follow-up. Providing medical information and tools is part of the support. Our study demonstrates that supporting means that the necessary guidance is provided or built up around a patient. In this environment, healthcare professionals and patients should act as equal partners. Fu et al. [41] also revealed the importance of patients’ partnerships with healthcare professionals on the ability to self-manage. Our interview participants expressed the desire to be involved in the entire care process. The challenge seems to shape this collaboration and define the contribution of the patient himself [15, 42]. A balance must be achieved between respecting patients’ autonomy and encouraging their active contribution in healthcare [43]. A recent systematic review on patient involvement identified factors that determine involvement in medical performance, namely the attitudes of the individual healthcare professional, the patient characteristics, the understanding of the purpose of involvement and the key relationships (i.e., doctor-patient relationship and the profession‐public relationship) [44]. According to our interview data,, the extent to which involvement contributes to self-management depends very much on the individual patient. Consequently, determining patients’ wishes and goals is an important component of self-management support. Several studies confirm this association with goal-oriented care [45, 46]. Because of the strong association, healthcare professionals are challenged to actively question patients to determine goals. Conversation is shaped using interrogation. This questioning aspect was strongly emphasised in the interviews. Open-ended questions enable physicians to obtain crucial and sufficient health information from patients [47]. An essential condition for this is that healthcare professionals commit to active listening. Unfortunately, healthcare professionals are used to telling, rather than actively questioning, what patients want or what feels good for them [48]. Miles et al. [49] confirms these findings by identifying communication as crucial for effective self-management. The study analyses patients’ experiences and reveals that patients expect to be listened to. Remarkable, our interview participants consider listening to patients as a natural one-way behaviour in providing good care, which also includes supporting self-management [50]. However, being in the position to listen to patients depends on various aspects. These aspects are not only related to the commitment and skills of healthcare professionals. In primary care practice, listening is additionally hampered by various factors, such as limited skills of patients and limiting contextual factors (e.g., doctor’s mood, workload, lack of time) [51, 52]. Moreover, asking question and listening are not stand-alone elements to facilitate dialogue [53]. Integrating moments of silence and paying attention to nonverbal communication significantly contributes to patient-centred communication [54–56]. In addition, dealing with patients’ cues and concerns is a foundation listening skill for GPs [51]. Unfortunately, there is evidence that these cues and concerns are not picked up or even ignored in daily primary care consultations [57, 58]. More training is needed to develop communication competences and skills of healthcare professionals beyond asking questions and offering a listening ear. To optimally deliver self-management support, a multidisciplinary approach is required. Tocchi et al. [59] described a positive effect of multidisciplinary teams on self-management of symptoms. Our research emphasises the role of a central healthcare professional to coordinate care. According to the interview data, the main actor in supporting self-management is context and patient dependent. Nevertheless, we can argue that in most cases the GP is mentioned as the pivotal figure. Unfortunately, not all GPs are able to fulfil this pivotal role [60, 61]. This must be considered when organising care around chronic patients. GPs must be taught the necessary competencies and skills in leadership [62].

Although our findings are in agreements with other studies regarding self-management support, most studies focus on one single aspect of the support. Few comprehensive models have been designed that include multiple aspects [20, 26, 27]. In addition, most models don’t target multimorbidity and only focus on self-management support from a specific healthcare professional [28]. Our SILCQ-model addresses this issue by providing insights into the fundamentals of support strategies, independent of the type of disease and healthcare professional. Self-management support interventions are not applicable to every patient and subject to change [63]. Therefore, it is important to consult the SILCQ-model which represents the foundation on which to build interventions.

### Strengths and limitations

Some limitations should be mentioned. First, patients were interviewed in the presence of their informal caregiver for additional support. This may have caused the participants withholding personal stories resulting in some bias. However, involving the care network of patients resulted in valuable insights. It should be noted that these insights only apply to patients. No conclusions about informal care can be drawn from this study. To be as comprehensive as possible, we continued to interview dyads until saturation of data was reached. Since in the last interviews, no new information on the topic of self-management support appeared, we decided the stop data collection after interviewing 16 dyads. Secondly, since participation in this research was completely voluntary, volunteer bias can occur. However, our research sample can be considered clinically representative in the international primary care context, when focusing on patients with complex care needs (i.e., mostly an aging population). Thirdly, the SILCQ-model is the result of discussions only with patients. Not including healthcare professionals allowed patients’ voice to be the focus of the model. Involving healthcare professionals should be the next step to validate the results. Fourthly, the SILCQ-model does not provide any recommendations for future self-management support interventions. We aimed to provide insights, rather than formulate specific guidelines because effectiveness of interventions is highly context and patient dependent. In addition, we believe that the SILCQ-components are the fundaments of every self-management support intervention in primary care. Lastly, the choice to conduct a qualitative content analysis limited the generalisability of the results. However, rather than generalising we aimed to undertake a proof of concept to understand the features of self-management support more in depth.

### Implications for research and practice

The SILCQ-model provides a holistic approach to self-management support, while focusing on the central interaction in the care network between healthcare professionals and patients. Programme developers are encouraged to keep the formulated elements in mind when setting up new self-management support interventions in primary care. Further research is required to understand how these SILCQ-elements are implemented in care practice and contribute to self-management outcomes.

## Conclusion

This qualitative paper highlights the importance of the SILCQ-elements – Supporting, Involving, Listening, Coordinating and Questioning – when reinforcing patients’ self-management. In providing good care, healthcare professionals are expected to prioritize these actions. The model should be further explored by all stakeholders to support the development of self-management interventions in primary care practice.

## Supplementary Information

**Additional file 1.**

## Data Availability

The datasets used and/or analysed during the current study available from the corresponding author on reasonable request. The interview guide is made available at: https://kuleuven-my.sharepoint.com/:b:/g/personal/lotte_timmermans_kuleuven_be/EVzVbmXRf3JEqRHNMVNEYFMBygAE0Vc380kvRrz2rRD0Iw?e=sfo6gI.

## Abbreviations

WHO: : World health organization
WISE: Whole system informing self-management engagement
PCA: Primary care academy
GP: General practitioner
SILCQ: Supporting, involving, listening, coordinating and questioning
