# Supplementary material for: Self-management support in flemish primary care practice: the development of a preliminary conceptual model using a qualitative approach
Source: BMC Prim Care. 2022 Mar 31;23:63. doi: 10.1186/s12875-022-01652-8 (PMC8968094; doi:10.1186/s12875-022-01652-8)
Supplement: Supplementary file 1 — Additional file 1. [file 12875_2022_1652_MOESM1_ESM.pdf]

Co-author	Position in Primary Care Academy	Affiliation	Faculty	Department	Adress	Email adress
Roy Remmen	Chair PCA	University of Antwerp	Faculty of Medicine and Health Sciences	Department of Primary Care and Interdisciplinary Care Antwerp	Doornstraat 331, 2610 Antwerp, Belgium	Roy.remmen@uantwerpen.be
Emily Verté	Coordinator and co-chair PCA, supervisor PhD student evaluation and implementation	University of Antwerp<br><br>Vrije Universiteit Brussel	Faculty of Medicine and Health Sciences<br>Faculty of Medicine and Pharmacy	Department of Primary Care and Interdisciplinary Care Antwerp<br>Department of Family Medicine and Chronic Care	Doornstraat 331, 2610 Antwerp, Belgium<br>Laarbeeklaan 103, 1090 Brussels	Emily.verte@vub.be
Muhammed Mustafa Sirimsi	PhD student interprofessional collaboration (IPC)	University of Antwerp	Faculty of Medicine and Health Sciences	General Practice	Doornstraat 331, 2610 Antwerp, Belgium	Muhammedmustafa.sirimsi@uantwerpen.be
Peter Van Bogaert	Lead work package IPC<br>Supervisor PhD student IPC	University of Antwerp	Faculty of Medicine and Health Sciences	Workforce Management and Outcome Research in Care	Universiteitsplein 1, 2610 Antwerp, Belgium	Peter.vanbogaert@uantwerpen.be
Hans De Loof	Supervisor PhD student IPC, member research team IPC	University of Antwerp	Faculty of Medicine and Health Sciences<br>Faculty of Pharmaceutical, Biomedical and Veterinary Sciences	Laboratory of Physiopharmacology	Universiteitplein 1, 2610 Antwerp, Belgium	Hans.deloof@uantwerpen.be
Kris Van den Broeck	Supervisor PhD student IPC, member research team IPC	University of Antwerp	Faculty of Medicine and Health Sciences	Department of Primary Care and Interdisciplinary Care Antwerp	Doornstraat 331, 2610 Antwerp, Belgium	Kris.vandenbroeck@uantwerpen.be
Sibyl Anthierens	Supervisor PhD student evaluation and implementation	University of Antwerp	Faculty of Medicine and Health Sciences	General Practice	Doornstraat 331, 2610 Antwerp, Belgium	Sibyl.anthierens@uantwerpen.be
Ine Huybrechts	PhD student evaluation and implementation	University of Antwerp	Faculty of Medicine and Health Sciences	General Practice	Doornstraat 331, 2610 Antwerp, Belgium	Ine.huybrechts@uantwerpen.be
Peter Raeymaeckers	Supervisor PhD student evaluation and implementation	University of Antwerp	Faculty of Social Sciences	Department of Sociology	Sint-Jacobstraat 2-4, 2000 Antwerp, Belgium	Peter.raeymaeckers@uantwerpen.be

Veerle Buffel	Member research team work package needs assessment and environmental scan	University of Antwerp	Faculty of Social Sciences	Department of Sociology; centre for population, family and health	Kipdorp 62, 2000 Antwerp, Belgium	Veerle.buffel@uantwerpen.be
Dirk Devroey	Member Brussels department PCA	Vrije Universiteit Brussel	Faculty of Medicine and Pharmacy	Department of Family Medicine and Chronic Care	Laarbeeklaan 103, 1090 Brussels, Belgium	Dirk.devroey@vub.ac.be
Bert Aertgeerts	Member work package self-management	Catholic University of Leuven	Faculty of Medicine	Academic Centre for General Practice, Department of Public Health and Primary Care	Kapucijnevoer 33, 3000 Leuven, Belgium	Bert.aertgeerts@kuleuven.be
Birgitte Schoenmakers	Lead work package self-management Supervisor PhD student self-management	Catholic University of Leuven	Faculty of Medicine	Academic Centre for General Practice, Department of Public Health and Primary Care	Kapucijnenvoer 33/7001, 3000 Leuven, Belgium	Birgitte.schoenmakers@kuleuven.be
Lotte Timmermans	PhD student self-management	Catholic University of Leuven	Faculty of Medicine	Academic Centre for General Practice, Department of Public Health and Primary Care	Kapucijnenvoer 33/7001, 3000 Leuven, Belgium	Lotte.timmermans@kuleuven.be
Veerle Foulon	Supervisor PhD student self-management	Catholic University of Leuven	Faculty of Pharmaceutical Sciences	Department of Pharmaceutical and Pharmacological Sciences	Herestraat 49, 3000 Leuven, Belgium	Veerle.foulon@kuleuven.be
Anja Declercq	Supervisor PhD student evaluation and implementation	Catholic University of Leuven	Faculty of Social Sciences	LUCAS- Centre for Care Research and Consultancy	Minderbroedersstraat 8, 3000 Leuven, Belgium	Anja.declerq@kuleuven.be
Nick Verhaeghe	Communication and valorization manager	Catholic University of Leuven – HIVA	Research Institute for Work and Society	Research group Social and Economic Policy and Social Inclusion	Parkstraat 47, 3000 Leuven, Belgium	Nick.verhaeghe@kuleuven.be
Dominique Van de Velde	Supervisor PhD student goal-oriented care	University of Ghent Artevelde University of Applied Scienes	Faculty of Medicine and Health Sciences	Department of Rehabilitation Sciences Department of Occupational Therapy	C. Heymanslaan 10, 9000 Ghent, Belgium	Dominique.vandevelde@ugent.be

Pauline Boeckxstaens	Co-lead work package goal-oriented care	University of Ghent	Faculty of Medicine and Health Sciences	Department of Public Health and Primary Care	C. Heymanslaan 10, 9000 Ghent, Belgium	Pauline.boeckxstaens@ugent.be
An De Sutter	Lead work package goal-oriented care	University of Ghent	Faculty of Medicine and Health Sciences	Department of Public Health and Primary Care	C. Heymanslaan 10, 9000 Ghent, Belgium	An.desutter@ugent.be
Patricia De Vriendt	Lead work package needs assessment and environmental scan<br>Supervisor PhD student goal-oriented care	Vrije Universiteit Brussel<br>University of Ghent<br>Artevelde University of Applied Scienes	Faculty of Medicine and Pharmacy	Department of Gerontology and Frailty in Ageing Research Group<br>Department of Rehabilitation Sciences<br>Department of Occupational Therapy		Patricia.devriendt@arteveldehs.be
Lies Lahousse	Member research team goal-oriented care	University of Ghent	Faculty of Pharmaceutical Sciences	Department of Bioanalysis	Ottergemsesteenweg 460, 9000 Ghent, Belgium	Lies.lahousse@ugent.be
Peter Pype	Member research team goal-oriented care, Member work package interprofessional collaboration	University of Ghent	Faculty of Medicine and Health Sciences	Department of Public Health and Primary Care<br>End-of-life Care Research Group	C. Heymanslaan 10, 9000 Ghent, Belgium	Peter.pype@ugent.be
Dagje Boeykens	PhD student goal-oriented care	University of Ghent	Faculty of Medicine and Health Sciences	Department of Rehabilitation Sciences<br>Department of Public Health and Primary Care	C. Heymanslaan 10, 9000 Ghent, Belgium	Dagje.boeykens@ugent.be
Ann Van Hecke	Member steering committee work package self-management	University of Ghent	Faculty of Medicine and Health Sciences	Department of Public Health and Primary Care/ University Centre of Nursing and Midwifery	C. Heymanslaan 10, 9000 Ghent, Belgium	Ann.vanhecke@ugent.be

Peter Decat	Co – supervisor PhD student self-management	University of Ghent	Faculty of Medicine and Health Sciences	Department of Public Health and Primary Care	C. Heymanslaan 10, 9000 Ghent, Belgium	Peter.decat@ugent.be
Rudi Roose	Member supervising committee work package goal-oriented care	University of Ghent	Faculty of Psychology and Educational Sciences	Department of Social Work and Social Pedagogy	Henri Dunantlaan 2, 9000 Ghent, Belgium	Rudi.roose@ugent.be
Sandra Martin	Lead work package education	University College Leuven-Limburg		Expertise Centre Health Innovation	Herestraat 49, 3000 Leuven, Belgium	Sandra.martin@ucll.be
Erica Rutten	Member advisory committee work package goal-oriented care	University College Leuven-Limburg		Expertise Centre Health Innovation	Herestraat 49, 3000 Leuven, Belgium	Erica.rutten@ucll.be
Sam Pless	Member research team needs assessment and environmental scan	University College Leuven-Limburg		Expertise Centre Health Innovation	Herestraat 49, 3000 Leuven, Belgium	Sam.pless@ucll.be
Vanessa Gauwe	Member research team interprofessional collaboration and education	Artevelde University of Applied Sciences		Department of Occupational Therapy	Voetweg 66, 9000 Ghent, Belgium	Vanessa.gauwe@arteveldehs.be
Didier Reynaert	Member research team self-management and education	University College of Applied Sciences Ghent		E-QUAL	Geraard de Duivelstraat 5, 9000 Ghent, Belgium	Didier.reynaert@hogent.be
Leen Van Landschoot	Member research team interprofessional collaboration and education	University of Applied Sciences College Ghent		Department of Nursing	Geraard de Duivelstraat 5, 9000 Ghent, Belgium	Leen.vanlandschoot@hogent.be

Maja Lopez Hartmann	Member research team needs assessment and environmental scan, interprofessional collaboration, education	Karel de Grote University of Applied Sciences		Department of Health Care	Brusselstraat 45, 2018 Antwerp, Belgium	Maja.lopezhartmann@kdg.be
Tony Claeys	Member research team education	VIVES University of Applied Sciences		LiveLab	Doorniksesteenweg 145, 8500 Kortrijk	Tony.claeys@vives.be
Hilde Vandenhoudt	Member of work package self-management, interprofessional collaboration, education	Thomas University of Applied Sciences, Kempen		LiCalab	Campus Blairon 800, 2300 Turnhout, Belgium	Hilde.vandenhoudt@thomasmore.be
Kristel De Vliegher	Member research team interprofessional collaboration, member advisory committee goal-oriented care	White-Yellow Cross		Nursing department – homecare	Frontipsiestraat 8, 1000 Brussels, Belgium	Kristel.de.vliegher@vlaanderen.wgk.be
Susanne Op de Beeck	Member of work package needs assessment and environmental scan, member advisory committee goal-oriented care	Flemish Patient Platform			Groenveldstraat 15, 3001 Heverlee, Belgium	Susanne.opdebeeck@vlaamspatientenplatform.be
